# New Insights on the Role of Urea on the Dissolution and Thermally-Induced Gelation of Cellulose in Aqueous Alkali

**DOI:** 10.3390/gels4040087

**Published:** 2018-12-11

**Authors:** Luis Alves, Bruno Medronho, Alexandra Filipe, Filipe E. Antunes, Björn Lindman, Daniel Topgaard, Irina Davidovich, Yeshayahu Talmon

**Affiliations:** 1 CQC, Department of Chemistry, University of Coimbra, 3004-535 Coimbra, Portugal; luisalves@ci.uc.pt (L.A.); filipe.eufrasio@gmail.com (F.E.A.); bjorn.lindman@fkem1.lu.se (B.L.); 2 Faculty of Sciences and Technology (MEDITBIO), University of Algarve, Campus de Gambelas, Ed. 8, 8005-139 Faro, Portugal; aifilipe@ualg.pt; 3 Fibre Science and Communication Network (FSCN), Mid Sweden University, SE-851 70 Sundsvall, Sweden; 4 Division of Physical Chemistry, Department of Chemistry, Center for Chemistry and Chemical Engineering, Lund University, SE-221 00 Lund, Sweden; daniel.topgaard@fkem1.lu.se; 5 Department of Chemical Engineering and the Russell Berrie Nanotechnology Institute (RBNI), Technion-Israel Institute of Technology, Haifa 3200003, Israel; irinad@technion.ac.il (I.D.); ishi@technion.ac.il (Y.T.)

**Keywords:** cellulose, gelation, urea, NaOH, microrheology, cryo-transmission electronic microscopy, polarization transfer solid-state NMR, hydrophobic interactions

## Abstract

The gelation of cellulose in alkali solutions is quite relevant, but still a poorly understood process. Moreover, the role of certain additives, such as urea, is not consensual among the community. Therefore, in this work, an unusual set of characterization methods for cellulose solutions, such as cryo-transmission electronic microscopy (cryo-TEM), polarization transfer solid-state nuclear magnetic resonance (PTssNMR) and diffusion wave spectroscopy (DWS) were employed to study the role of urea on the dissolution and gelation processes of cellulose in aqueous alkali. Cryo-TEM reveals that the addition of urea generally reduces the presence of undissolved cellulose fibrils in solution. These results are consistent with PTssNMR data, which show the reduction and in some cases the absence of crystalline portions of cellulose in solution, suggesting a pronounced positive effect of the urea on the dissolution efficiency of cellulose. Both conventional mechanical macrorheology and microrheology (DWS) indicate a significant delay of gelation induced by urea, being absent until ca. 60 °C for a system containing 5 wt % cellulose, while a system without urea gels at a lower temperature. For higher cellulose concentrations, the samples containing urea form gels even at room temperature. It is argued that since urea facilitates cellulose dissolution, the high entanglement of the cellulose chains in solution (above the critical concentration, C*) results in a strong three-dimensional network.

## 1. Introduction

Aqueous solutions of sodium hydroxide, NaOH(aq.), are considered a favorable solvent for cellulose, mainly because they are inexpensive and have low toxicity [1]. The conditions for cellulose dissolution in NaOH(aq.) must be rigorously controlled: dissolution occurs within narrow temperature (i.e., sub-zero) and concentration (ca. 1.5–2.5 M) ranges [1,2]. The extent of cellulose dissolution is very dependent not only on the solvent used, but also on the conditions [2,3,4]. This is valid for NaOH(aq.) and for all the other solvent systems where dissolution might be complete, down to the molecular level, or incomplete, with fibrils or cellulose colloidal aggregates remaining in solution [5]. Consequently, the way cellulose is dissolved has consequences, not only in the rheology of the dopes (which may also affect other processes, such as fiber spinning), but also on the final properties of the generated materials [2,6].

The characterization of cellulose solutions is not trivial. This is mainly due to the complexity of most of the solvents used [7,8]. In this respect, polarization transfer solid-state nuclear magnetic resonance (PTssNMR) has been recently shown to be a very promising technique for efficient and robust characterization of the solution state of cellulose [2,3,9]. However, in order to have a reasonable signal/noise ratio, PTssNMR is currently limited to concentrated cellulose solutions (ca. 10 wt %). Alternatively, techniques like cryo-TEM (cryogenic-temperature transmission electron microscopy) can be applied in dilute conditions [10]. The use of this technique in cellulose solutions is still very limited, mainly due to the technical difficulties of properly vitrifying aqueous solutions containing high concentrations of salt and other complex additives, as often found in the majority of cellulose solvents, and issues of image contrast. A successful case has been introduced by Rein et al., who have reported images of microcrystalline cellulose (MCC) dissolved in an ionic liquid/cosolvent system [10].

Once dissolved, cellulose dopes are usually metastable and quite sensitive to aging or changes in the pH, polarity, or temperature of the medium [11,12,13]. Consequently, cellulose liquid dopes are prone to form gels, which represent an additional problem for industrial applications. Such gelation of cellulose solutions is a process not only interesting from a fundamental point of view, but of foremost importance for the development of cellulose-based films, hydrogels, fibers, etc. However, the gelation of cellulose dopes is still a poorly understood phenomenon, although it has been shown that the use of certain additives, such as urea or thiourea, improve the solution features [14]. Cellulose aggregation is minimized and the solutions formed in the presence of such additives become kinetically stable for several days [15,16]. The mechanism of action of these additives is not consensual, but it has been suggested that cellulose may form some sort of inclusion complexes with the additive, which are beneficial for molecular dissolution [14,17,18,19,20,21]. Other studies do recognize that urea promotes the decrease of crystallinity, enhancing the dissolved fraction of cellulose and the thermal stability of the dopes; however, surprisingly urea apparently has no direct interaction with cellulose [22,23]. In an opposite view, Hagman et al. found that the addition of urea has no significant effect on the dissolution of cellulose [24].

Urea is known as a good solubilising agent for organic molecules, such as proteins, and the mechanism has been suggested to occur via weakening of the hydrophobic interactions [25,26]. This is particularly relevant in the context of cellulose solubility, since hydrophobic interactions have been argued to strongly contribute to the insolubility pattern of cellulose [7,27,28,29,30,31,32].

In order to shine light on the dissolution and gelation of cellulose solutions and on how urea affects it, an unusual set of methods (PTssNMR, diffusion wave spectroscopy (DWS), and cryo-TEM) was used. To the best of our knowledge this is the first time microrheology and cryo-TEM images of MCC in NaOH(aq.) are reported. Additionally, cellulose gelation was also evaluated by common macrorheology (mechanical rheometer).

## 2. Results

**Figure 1:**
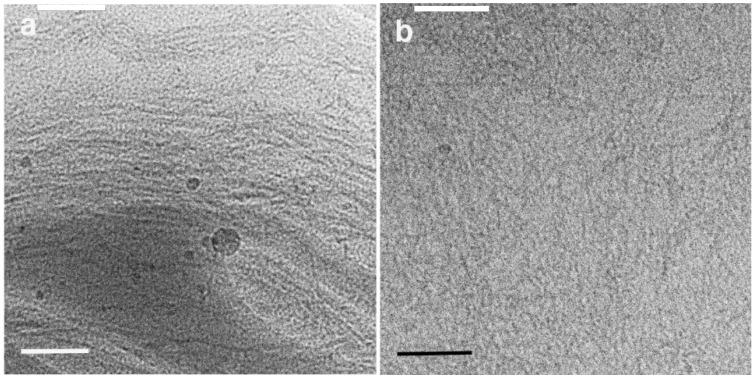
Cryo-transmission electronic microscopy (cryo-TEM) images of 0.5 wt % MCC dissolved in (**a**) 8 wt % NaOH(aq.) solution and (**b**) in 8 wt % NaOH(aq.)/12 wt % urea system. Scale bars correspond to 100 nm.

Cryo-TEM is a powerful technique that may provide relevant information about the dissolution state of cellulose. In Figure 1, successful cryo-TEM images from MCC dissolved in NaOH(aq.) with and without urea can be seen. It is observed that the NaOH(aq.) partially dissolves cellulose. Only some long fibrils and insoluble crystalline aggregates remain (Figure 1a). The addition of urea clearly improves dissolution, reducing the undissolved fraction of cellulose in solution (Figure 1b). Figure 1b also suggests the decrease of crystallinity, since the cellulose clusters look amorphous. The improvement of cellulose dissolution and decrease in crystallinity is in agreement with previous findings [22].

**Figure 2:**
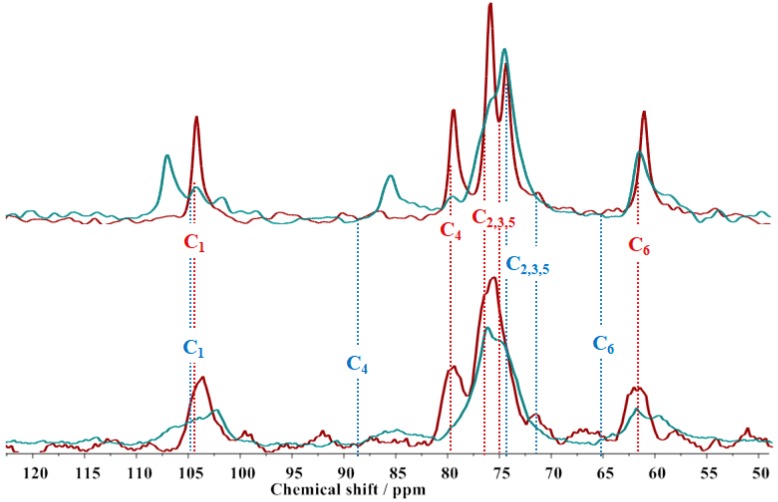
Polarization transfer solid-state nuclear magnetic resonance (PTssNMR) spectra for 10 wt % MCC dissolved in 8 wt % NaOH(aq.), with (**bottom**) and without (**top**) urea. The cross-polarization (CP, blue line) and the insensitive nuclei enhanced by the polarization transfer signal (INEPT, red line) spectra were acquired at 25 °C. The vertical lines represent the chemical shifts of native dry microcrystalline cellulose (MCC; blue dotted lines) [2] and dissolved cellulose [33] (red dotted lines).

To complement the cryo-TEM, PTssNMR was performed on concentrated cellulose samples. In Figure 2, the PTssNMR spectra of 10 wt % MCC dissolved in NaOH(aq.) with (bottom) and without (top) urea are depicted.

The MCC sample dissolved in an NaOH(aq.) solution, with and without urea, gives both CP (blue line) and INEPT (red line) signals. While the INEPT peaks of cellulose dissolved in NaOH(aq.) solution present chemical shifts that fit well with those assigned to the dissolved fraction of cellulose in ultra-centrifuged NaOH/D2O samples [33], the CP spectrum presents chemical shifts significantly different compared with solid MCC samples [2,9] (blue dotted vertical lines). A comparison of the chemical shifts obtained for the solid portion of cellulose in the NaOH(aq.) solution [107.0/104.2/101.7 (C1), 85.5 (C4), 74.6 (C2,3,5), and 61.7 (C6)], with the chemical shifts found in literature, (e.g., 106.9/ 104.2 (C1), 85.5/83.0 (C4) and 61.3 (C6)) suggests that cellulose is organized as the so called ‘‘Na-cellulose Q″, which is a highly swollen form of cellulose [34]. Additionally, the NaOH(aq.) sample presents a well-defined CP spectrum (good signal-to-noise ratio), which is indicative of a significant amount of undissolved material in solution.

When urea is present, (Figure 2 bottom), the CP and the INEPT signals are also detected, suggesting that dissolution is also not complete. However, the ratio between the CP and INEPT peaks is different. Even if neither CP nor INEPT are truly quantitative, the relationship between the intensity of the peaks is indicative of the MCC undissolved/dissolved ratio in solution. For the NaOH(aq.) without urea, the CP and INEPT peaks are of similar intensity, while when urea is added, the INEPT peaks are clearly more intense than the CP peaks, suggesting that the additive improves the dissolution efficiency. Moreover, the chemical shifts obtained for the solid fraction of cellulose (CP signal) in the NaOH(aq.)/urea system, (104.3/102.7 (C1), 84.8 (C4), 76.0/74.9 (C2,3,5) and 61.3/59.2 (C6)), can be assigned to an amorphous structure, deduced mainly from the chemical shifts of C1 and C4, which appear in an “amorphous zone” of the spectrum; and the broadening of the peaks, that is due to the broad distribution of the chemical shifts, results of the low ordered structure [35]. Overall, the nuclear magnetic resonance (NMR) data are in agreement with the cryo-TEM results; the presence of urea not only results in the decrease of the amount of the solid fraction present in solution, but also affects the remaining crystalline organization.

**Figure 3:**
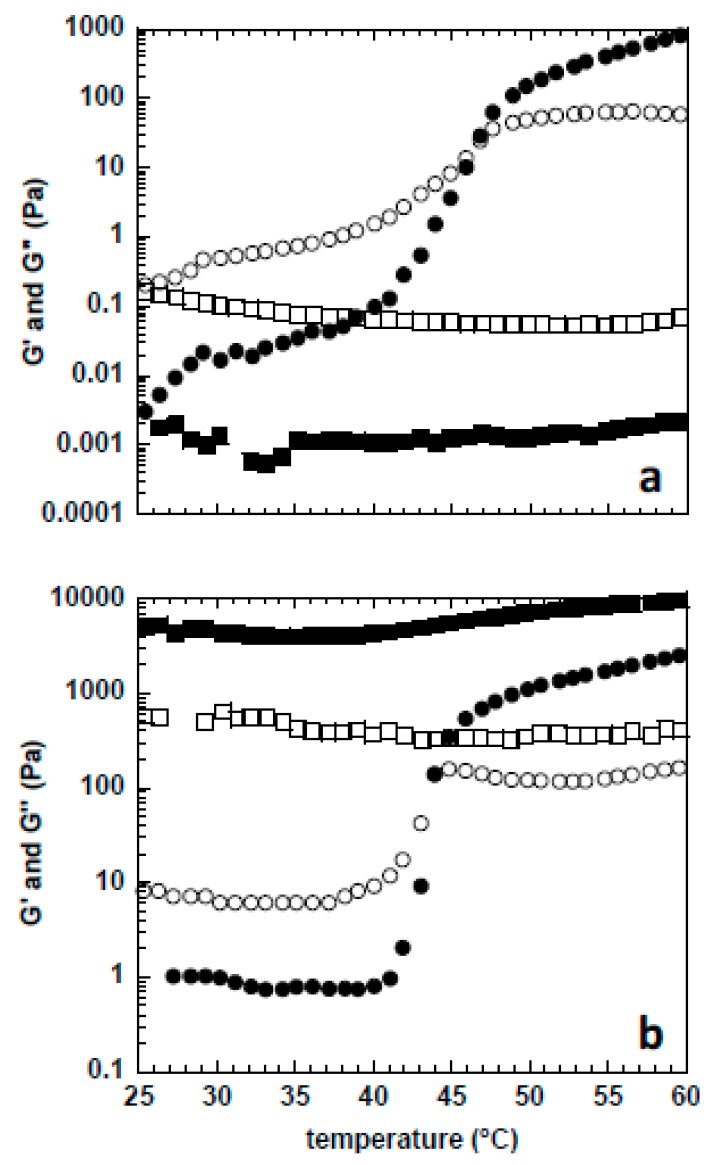
Viscoelastic parameters determined by mechanical rheometry. G′ (full symbols) and G″ (empty symbols) were obtained for samples with (squares) and without (circles) urea after a temperature sweep from 25 to 60 °C (heating rate of 1 °C/min) at a constant shear stress of 10 Pa and frequency of 0.1 Hz. (**a**) 5 wt % MCC and (**b**) 10 wt % MCC dissolved in 8 wt % NaOH(aq.)/12 wt % urea.

Solutions containing cellulose dispersed at the molecular level, or close to it, present rheological features quite different from those where undissolved fibrils or cellulose aggregates are still present. In this respect, macrorheology and microrheology were performed to better understand the rheological properties of cellulose in NaOH(aq.), and how urea affects it. In Figure 3, the viscoelastic parameters G′ and G″ are shown after temperature sweeps from 25 to 60 °C.

The sample containing 5 wt % MCC in NaOH (aq.) (Figure 3a) is liquid-like (G″ > G′) below ca. 46 °C, while it presents a solid-like behaviour (G′ > G″) above this temperature. Strikingly, the addition of 12 wt % urea delays the gelation point considerably, and the sample is always liquid-like (G″ > G′) up to 60 °C, the highest temperature studied. Likewise, the sample with 10 wt % MCC in NaOH (aq.) (Figure 3b) also presents a transition from liquid-to-solid like behaviour at a slightly lower temperature (i.e., 44 °C instead of 46 °C, as observed for the 5 wt % MCC). This gelation at a lower temperature is expected to be a concentration effect, where more cellulose interactions and physical entanglements can be established and contribute to an effective three-dimensional (3D) gel network at higher MCC concentrations.

It is well established that the stability of cellulose solutions in aqueous alkali is strongly temperature dependent. It has been found that the gelation time, estimated from the G′ and G″ crossover, decreases exponentially with increasing temperature [12,36]. Nevertheless, the reason for such gelation is still not clear [1,12,15]. Recently it has been suggested that the observed gelation is due to the precipitation and crystallization of cellulose. The crystallites may thus compete for the same cellulose molecules, and act as effective cross-linkers to form the 3D gel network [36]. In other work, Isobe et al. suggests that upon heating or using a coagulant, cellulose regeneration (gelation) begins by the hydrophobic stacking of monomolecular sheets, followed by their mutual association via hydrogen bonding [11]. This is clear evidence in favor of cellulose self-aggregation in the solution, due to its amphiphilicity. The progressively increased number of hydrophobic junction zones between cellulose chains (and cellulose crystallites) promoted by a temperature increase can be mitigated using appropriate additives, such as urea, capable of reducing the hydrophobic interactions responsible for cellulose aggregation [22,37]. As a consequence, these additives may improve the thermal stability of the solution. Thus, we believe that this is the main reason why the 5 wt % MCC solution does not gel up to 60 °C when urea is present; the dope stabilization may be due to the local accumulation of urea on the hydrophobic surface, preventing the hydrophobic association of dissolved cellulose molecules and leading to a liquid-like solution [22,37]. Interestingly, the 10 wt % MCC in NaOH (aq.)/urea presents a solid-like behaviour in all of the studied temperature range. As the cryo-TEM and PTssNMR data suggest, the addition of urea leads to a better dissolution of MCC. Consequently, this results in a higher effective number of cellulose chains molecularly dispersed in solution, and an expected increase of number of entanglements between cellulose chains. Therefore, the solution behaves always like a gel (G′ > G″) in the presence of urea.

The same behaviour could be anticipated for the 5 wt % MCC with urea sample, but since the overlap concentration has been estimated to be around ca. 4.8 wt % [24], we believe that the attractive interactions between cellulose chains are weakened by urea, and a robust gel is never formed. The sol-gel transition of the solutions in NaOH(aq.)/urea is accompanied by a large increase in the viscoelastic parameters when the MCC concentration goes from 5 to 10 wt %; the G′ value of the 10 wt % MCC system is about seven orders of magnitude higher than that of the 5 wt % MCC system. Conversely, in the systems without urea, the G′ increase is about four orders of magnitude lower at temperatures below the gelation temperature (*Tg*). Such a remarkable difference can also be explained by the improved dissolution of MCC in the presence of urea and the consequent increased number of cellulose entanglements in solution.

**Figure 4:**
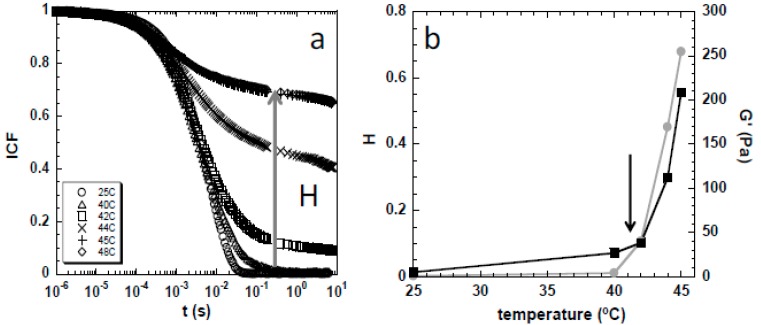
(**a**) Intensity correlation function (ICF) obtained with increasing temperature for the solution of 5 wt % MCC in NaOH(aq.), and (**b**) the corresponding H (*t* = 1 s; grey circles) and G′ (ω = 100 rad/s; black squares) as a function of temperature. The arrow on the left indicates how the ICF plateaus (and the H parameter) evolve with temperature rise, while the arrow on the right indicates the inflection point (*Tg*), where both the H parameter and G′ start increasing exponentially.

The thermal gelation of cellulose solutions in NaOH(aq.) and NaOH(aq.)/urea was also evaluated by DWS, and compared to the mechanical rheometry data. DWS is an optical technique that extracts the rheological information from the sample based on the Brownian motion of particle tracers (see [36] and references therein). The use of DWS for at-line measurements allows estimation of *Tg* since the gelation point is defined by the moment where the normalized intensity correlation function (ICF) no longer decays to zero [38]. This is exemplified for the 5 wt % MCC in NaOH(aq.), and is displayed in Figure 4a. At 25 °C, the ICF decays to zero, but at 40 °C this is no longer observed. The inability of the ICF to fully decay to zero is a consequence of the particle movement restriction, suggesting that the onset of gelation occurs at a *Tg* of ca. 40 °C. To better evaluate variations in the ICF decay with temperature, the H parameter, corresponding to the plateau of the ICF curve at long lag times (ca. *t* = 1 s) [38], was determined and plotted as a function of temperature (Figure 4b). The H parameter should be interpreted as follows: when H equals to zero, it indicates that the particles movement is free at long observation times and the sample has a liquid-like behavior; on the other hand, if H is higher than zero, it reflects the existence of a restriction in the tracer′s movements at long observation times, which is characteristic of a gel-like structure. The H parameter progressively increases above ca. 40 °C, and since the elastic modulus (G′) follows the same trend (Figure 4b), it suggests that the gel structure is formed and strengthened upon heating above *Tg*.

**Figure 5:**
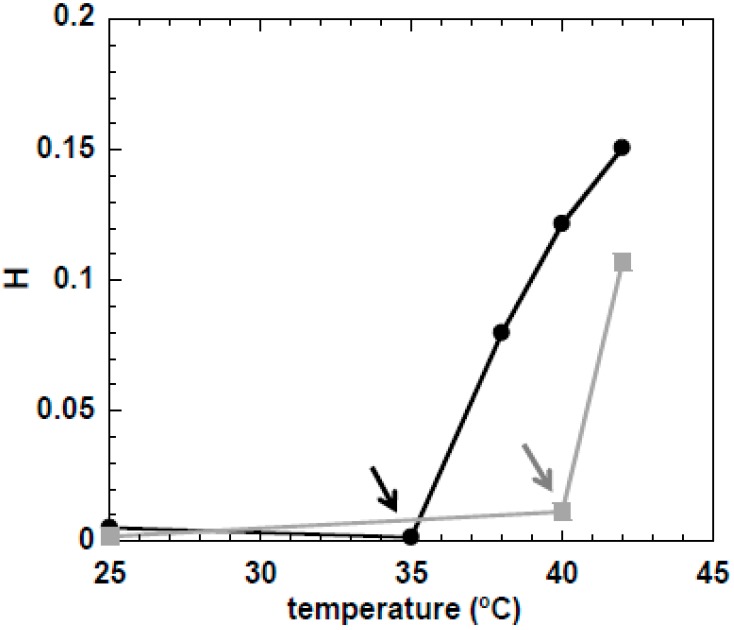
Variation of the H parameter with temperature for the 5 wt % (grey squares) and 10 wt % (black circles) MCC samples dissolved in NaOH(aq.). The arrows indicate the *Tg*.

In order to evaluate the effect of cellulose concentration on *Tg*, the 10 wt % MCC in NaOH(aq.) was also analyzed, and the variation of H with temperature was compared to that of the sample of 5 wt % MCC. As can be seen in Figure 5, the accentuated increase of H starts at ca. 35 °C for the 10 wt % MCC, and 5 °C earlier than its counterpart 5 wt % MCC.

The higher the MCC concentration, the lower *Tg* is. Remarkably, when cellulose concentration is increased, a shift to a lower *Tg* was qualitatively observed by both techniques. The slight difference in the estimated *Tg* values between the two techniques may be attributed to the distinctive principles and approaches that underlie each method and some artefacts induced by tracer–tracer or tracer–polymer interactions [39].

**Figure 6:**
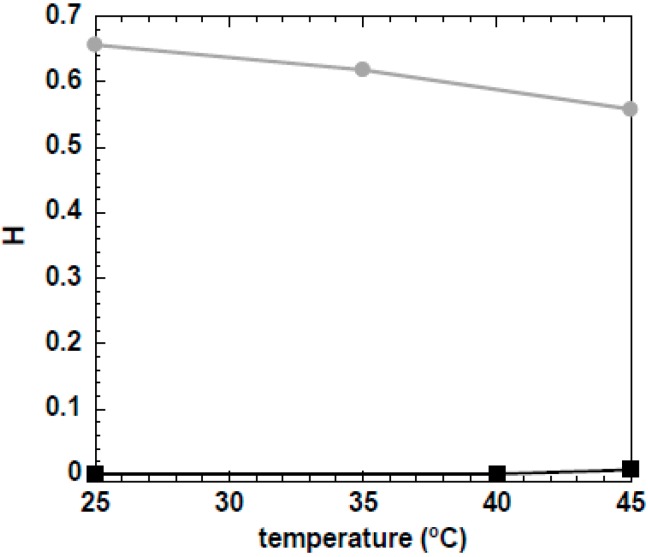
Variation of the H parameter with temperature for the 5 wt % (black squares) and 10 wt % (grey circles) MCC samples dissolved in 8 wt % NaOH(aq.)/12 wt % urea.

The solutions composed of MCC dissolved in NaOH(aq.)/urea were also studied by DWS, and the H profile with temperature is represented in Figure 6. As can be observed, the H parameter of the 5 wt % MCC is approximately zero, with no significant variations in the studied temperature range, which suggests free movement of tracer particles and thus a liquid-like system. Conversely, the 10 wt % MCC behaved as a strong gel during the temperature range, with corresponding high H values (incomplete ICF decay), thus reflecting a very high restriction in the movement of the tracers embedded in the gel network.

These results support the findings presented from mechanical rheology, showing the ability of urea to improve cellulose dissolution by reducing the hydrophobic interactions necessary to trigger gelation, resulting in higher thermal stability, as observed for the 5 wt % MCC sample. However, as the MCC concentration is increased, more molecularly dissolved cellulose, which is prone to entangle, will be present in solution, resulting in a stronger gel network.

## 3. Conclusions

The effect of urea on the dissolution and gelation of cellulose in aqueous alkali was revisited, employing an unusual set of advanced methods. Cellulose fibrils and microcrystallites were found in the NaOH(aq.) systems, while the addition of urea led to a decrease of aggregates in solution and absence of crystallites. Cryo-TEM data strongly suggests an improvement in the dissolution performance of the alkali solvent with the introduction of urea, which is known to weaken hydrophobic interactions. A similar trend was observed in PTssNMR, where no crystalline portions of cellulose were detected in the NaOH(aq.)/urea system. PTssNMR gives direct discrimination between the dissolved and undissolved states based on the rate of C–H bond reorientation. Dissolution in highly viscous media can lead to reorientational correlation times exceeding 10 ns, leading to an enhancement of the CP signal, even if the sample is well dissolved. However, the CP signal from well dissolved samples is typical of an amorphous arrangement, as obtained with NaOH(aq.)/urea system.

Macro- and microrheology data suggests that the thermal gelation of cellulose dissolved in NaOH(aq.) is delayed in the presence of urea (i.e., *Tg* is shifted to a higher temperature). Conversely, cellulose concentration was also found to affect the gelation process. While for the semi-dilute solutions of cellulose (ca. 5 wt %), urea completely prevents gelation, due to its ability to reduce the aggregation of cellulose chains, for cellulose concentrations well above C* a gel is obtained in the entire temperature range studied. In the latter case, gelation of the cellulose solution is promoted by improvement of the entanglement of cellulose chains, since urea is beneficial for dissolution by increasing the amount of molecularly dissolved cellulose in the medium.

Overall, cellulose dissolution in aqueous alkali solutions is found to be improved by urea. This additive remarkably affects the solution stability, preventing thermal gelation in certain conditions. It is suggested here that the urea effect on dissolution and gelation process is driven by its capacity to weaken hydrophobic interactions in amphiphilic-like compounds, such as cellulose. These results improve our general understanding of cellulose in aqueous alkali solutions, and contribute to supporting the role of hydrophobic interactions on cellulose insolubility.

## 4. Materials and Methods

### 4.1. Materials

Microcrystalline cellulose Avicel PH-101, with an average particle size of 50 µm and degree of polymerization of ca. 260, and urea (99.5% purity) were acquired from Sigma-Aldrich (St. Louis, MO, USA), while NaOH pellets (98% purity) were obtained from Fluka (Fisher Scientific, Leicestershire, UK). All chemicals were used as received. The water used was purified in-house using a MILLIPORE Milli-Q Gradient A10 system from Millipore (Burlington, MA, USA).

### 4.2. Methods

#### 4.2.1. Sample Preparation

Cellulose dissolution was achieved by adapting a known procedure reported elsewhere [2,36]. Briefly, the alkali-based systems were prepared by the addition of the desired amount of cellulose (0.5, 5, or 10 wt %) and urea (12 wt % when used), in an 8 wt % NaOH/H_2_O solution, which was then allowed to freeze at −18 °C for 2 h. This was followed by thawing the solid frozen mass at room temperature with simultaneous vigorous mixing.

#### 4.2.2. Polarization Transfer Solid-State Nuclear Magnetic Resonance

NMR experiments were performed on a Bruker AVII-500 spectrometer with a 4 mm ^13^C/^31^P/^1^H E-free probe (Bruker, Hamburg, Germany), running at the ^1^H and ^13^C Larmor frequencies of 500 and 125 MHz, respectively. The spectra were recorded at 5 °C and 25 °C, using 4 mm HR-MAS rotors (Bruker, Germany) specifically designed for retaining liquids during magic angle spinning (MAS) at a spinning frequency of 5 kHz. The temperature was varied using a BVT-2000 temperature control, and the spectra were acquired under 88 kHz, two-phase pulse modulation (TPPM) decoupling, using a 20 ms acquisition time with 300 ppm spectral width and an 80 kHz nutation frequency for the 90° and 180° pulses. Cross-polarization (CP) was performed with CP = 1 ms and 80 kHz ^13^C nutation frequency linearly ramped from 72 to 88 kHz ^1^H nutation frequency. The time delays of τ = 1.8 ms and τ′ = 1.2 ms were used to refocus the insensitive nuclei enhanced by the polarization transfer signal (INEPT). The ^13^C spectra were externally referenced to alpha-glycine at 43.67 ppm (α carbon). The pulse sequence schemes that together constitute PTssNMR follow the previous scheme reported by Gustavsson et al. [9]. The theoretical CP and INEPT efficiencies as a function of the rate and anisotropy of C–H bond reorientation also follow the scheme presented by Gustavsson et al. [9]. This approach demonstrates potential for the detailed characterization of both the liquid and the solid phases in cellulose dissolution media [2,3]. Through the ^13^C chemical shifts, the PTssNMR method may give information about chain packing, molecular structure, and conformation. It also provides information about molecular dynamics by means of the signal intensities obtained with the polarization transfer schemes CP and INEPT [9].

#### 4.2.3. Cryo-Transmission Electronic Microscopy

For cryo-specimen preparation, a small drop (ca. 3 µL) of the sample solution was applied on a perforated carbon film supported on a copper transmission electronic microscopy (TEM) grid (Lacey Formvar/carbon films on 200 mesh Cu grids, Ted Pella, Redding, CA, USA), held by tweezers inside a controlled environment vitrification system (CEVS; [40]). To achieve good wettability of the support, the perforated films were first cleaned with glow-discharge air-plasma in a PELCO EasiGlow system (Ted Pella). The CEVS was kept at 25 °C and 100% air water saturation. After the drop was placed onto the grid and blotted, the grid was immediately plunged quickly into liquid ethane at its freezing point of −183 °C. The specimens were examined in an FEI Talos 200C, FEG-equipped HR-TEM, operating at 200 kV while being kept below −176 °C in a Gatan 626 cryo-specimen holder (Thermo Fisher Scientific, Hillsboro, OR, USA). Specimens were studied in the low-dose imaging mode to minimize electron beam exposure and radiation damage. Images were recorded digitally by an FEI Flacon III direct-imaging, 4 k × 4 k pixel, camera (Thermo Fisher Scientific). To enhance image contrast, we used a Volta “phase plate”, a device that converts phase differences into amplitude difference, analogous to the phase-plate in light microscopy [41].

#### 4.2.4. Mechanical Rheometry

The rheological measurements were carried out on a HAAKE MARS III rheometer (Thermo Fisher Scientific, Karlsruhe, Germany) set with plate–plate geometry (35 mm, 0.2 mm gap). A Peltier unit was used to ensure strict temperature control, which was varied from 5 to 60 °C at a constant rate of 0.015 °C/s. A solvent trap was used to prevent water evaporation. The storage modulus (G′) and the loss modulus (G″) were accessed by performing dynamic oscillatory experiments at 0.1 Hz, with a constant stress of 10 Pa.

#### 4.2.5. Diffusion Wave Spectroscopy

Diffusing wave spectroscopy (DWS) assays were performed with the DWS RheoLab (LS Instruments AG, Fribourg, Switzerland) equipped with the echo technology and configured to automatically estimate the transport mean free path, as described by Zhang et al. [42]. The transmission mode was used to access the microrheological properties of the samples. DWS is a powerful optical technique suitable to investigate turbid samples in a nondestructive and reproducible way, providing information on the static and dynamic rheological properties of the system [43]. Polystyrene latex particles with high surface density of carboxyl groups and with a mean size of 400 nm (PL6204-6101, Agilent Technologies, Santa Clara, CA, USA) were used as tracers, and added to the sample solution to a final concentration of 0.5% (*w*/*w*). The samples were analyzed in cuvettes with 10 mm of thickness after being equilibrated for 900 s in the measuring chamber at the desired temperature. Assays were performed from 25 to 45 °C, and the measurement time was adjusted to obtain the complete intensity correlation function (ICF) curve until a lag time of 20 s.
